# Health status in the TORCH study of COPD: treatment efficacy and other determinants of change

**DOI:** 10.1186/1465-9921-12-71

**Published:** 2011-05-31

**Authors:** Paul W Jones, Julie A Anderson, Peter MA Calverley, Bartolome R Celli, Gary T Ferguson, Christine Jenkins, Julie C Yates, Jørgen Vestbo, Michael D Spencer

**Affiliations:** 1 Department of Cardiac and Vascular Sciences, St George's Hospital, University of London, London, UK; 2 Respiratory Medicine Development Centre, GlaxoSmithKline, Greenford, UK; 3 School of Clinical Science, University Hospital Aintree, Liverpool, UK; 4 Pulmonary Division, Brigham and Women's Hospital, Harvard Medical School, Boston, MA, USA; 5 Pulmonary Research, Institute of Southeast Michigan, Livonia, MI, USA; 6 Clinical Management Research Group, Woolcock Institute of Medical Research, Sydney, Australia; 7 Respiratory Medicine Development Center, GlaxoSmithKline, Research Triangle Park, NC, USA; 8 North West Lung Centre, Wythenshawe Hospital, Manchester, UK and Department of Cardiology & Respiratory Medicine, Hvidovre Hospital, Hvidovre, Denmark; 9 Respiratory Medicine Development Centre, GlaxoSmithKline, Greenford, UK; 10 Janssen Cilag Ltd, UK

**Keywords:** COPD, quality of life, health status, lung function

## Abstract

**Background:**

Little is known about factors that determine health status decline in clinical trials of COPD.

**Objectives:**

To examine health status changes over 3 years in the TORCH study of salmeterol+fluticasone propionate (SFC) vs. salmeterol alone, fluticasone propionate alone or placebo.

**Methods:**

St George's Respiratory Questionnaire (SGRQ) was administered at baseline then every 6 months.

**Measurements and Main Results:**

Data from 4951 patients in 28 countries were available. SFC produced significant improvements over placebo in all three SGRQ domains during the study: (Symptoms -3.6 [95% CI -4.8, -2.4], Activity -2.8 [95% CI -3.9, -1.6], Impacts -3.2 [95% CI -4.3, -2.1]) but the pattern of change over time differed between domains. SGRQ deteriorated faster in patients with Global Initiative for Chronic Obstructive Lung Disease (GOLD) stages III & IV relative to GOLD stage II (p < 0.001). There was no difference in the relationship between deterioration in SGRQ Total score and forced expiratory volume in one second (FEV_1_) decline (as % predicted) in men and women. Significantly faster deterioration in Total score relative to FEV_1 _% predicted was seen in older patients (≥ 65 years) and there was an age-related change in Total score that was independent of change in FEV_1_. The relationship between deterioration in FEV_1 _and SGRQ did not differ in different world regions, but patients in Asia-Pacific showed a large improvement in score that was unrelated to FEV_1 _change.

**Conclusions:**

In addition to treatment effects, health status changes in clinical trials may be influenced by demographic and disease-related factors. Deterioration in health status appears to be fastest in older persons and those with severe airflow limitation.

**Trial Registration:**

ClinicalTrials.gov: NCT00268216

## Background

The ability to reliably measure health status (sometimes referred to as health-related quality of life) by administering standardized questionnaires has greatly expanded our understanding of the effects of chronic respiratory diseases like chronic obstructive pulmonary disease (COPD) [1]. Disease-specific questionnaires like the St George's Respiratory Questionnaire (SGRQ) reflect a wide range of different health impacts in COPD [1], are designed to provide an overall measure of impairment, and are now used widely in randomized controlled trials in COPD. A number of relatively small longitudinal observational studies [2-5] have shown that a decline in health status may be seen over time, but there have been relatively few studies of long-term treatment effects on health status decline. The first of these was the Inhaled Steroids in Obstructive Lung Disease (ISOLDE) study, which showed that health status measured using the SGRQ deteriorated progressively over 3 years [6], an effect that was slowed by the inhaled corticosteroid (ICS) fluticasone propionate (FP) [7,8]. The recent 3-year Towards a Revolution in COPD Health (TORCH) and 4-year Understanding Potential Long-term Impacts on Function with Tiotropium (UPLIFT) studies have both reported health status gains that lasted the entire duration of the study [9,10]. In view of the pivotal nature of these studies, it is important to understand the nature of these improvements and factors that may influence them. We have used data from the TORCH trial to explore these factors.

TORCH was a double-blind placebo-controlled randomized parallel group study to investigate the benefits of FP and the long-acting beta_2 _agonist salmeterol (SAL) combined in one inhaler (SAL + FP [SFC]) vs. placebo [9]. Patients were recruited from 42 countries. The primary endpoint was all-cause mortality at 3 years, measured in the intention-to-treat (ITT) population. Health status, measured using the SGRQ, was a pre-specified secondary endpoint. The results obtained with the total SGRQ score have been reported [9]. In this analysis, we provide data about the effect of therapy on the SGRQ domains, and an analysis of demographic and disease-related factors that may influence long-term changes in health status.

## Methods

Details of the TORCH study design and analysis plan have been published previously [11]. The study was approved by local ethics review committees and conducted in accordance with the Declaration of Helsinki and Good Clinical Practice guidelines. All patients gave written informed consent. Methods pertaining specifically to the current analysis will be described here.

### Patients

The population for this study (the 'health outcomes population') was a subset of the total efficacy population. It consisted of patients for whom SGRQ questionnaire translations were determined to be linguistically and culturally valid, could potentially have a total SGRQ score calculated, and had completed at least one questionnaire during the study. Suitable translations were not available for five languages at the start of the study. Furthermore, where translations were available, during the years from study inception to conclusion the standards required for linguistic validity had evolved. We wished to ensure that all translations met current standards, so prior to breaking the treatment code, and independently of the sponsor, each country-language combination went through a process of two back translations, pilot testing in COPD patients and developer review. Based on this, one of three actions was taken:

• country-language combination was judged valid (n = 28)

• country-language combination was valid after developer-agreed modification of the scoring algorithm:

◦ if there were ≤ 2 poorly translated items that could be removed (n = 4)

◦ incorrect response ordering that could be corrected when scoring (n = 1)

• country-language combination was excluded - failed to meet current standards (n = 4).

For the analysis of the relationship between the change in forced expiratory volume in one second (FEV_1_) and the change in SGRQ, only patients with measurements of both endpoints during treatment and at the same timepoint were included. Therefore this is a subset of the general health outcomes population.

### Statistical analyses

Missed SGRQ items were handled according to the developers' instructions in the SGRQ manual. Scores were analyzed as change from baseline using mixed model repeated measures (MMRM) including treatment, smoking status, age, gender, baseline FEV_1_, body mass index (BMI), region, visit, treatment by visit, baseline SGRQ score, and visit by baseline SGRQ. Estimated treatment differences at each visit were averaged with equal weights to obtain the overall treatment effect over the study period.

The impact of the Global Initiative for Chronic Obstructive Lung Disease (GOLD) stage on change in SGRQ score from baseline was assessed using MMRM analysis of the placebo arm employing identical covariates, except that the GOLD stage was incorporated into the model and treatment was not.

The impact of demographic factors on the relationship between change in SGRQ and change in FEV_1 _over 3 years was tested using analysis of covariance, with and without adjustment for baseline covariates. All treatment arms were combined for this analysis; because of its exploratory nature, significance was accepted at p < 0.01. We tested both the slope of this relationship and the intercept, this being the change in SGRQ associated with no change in FEV_1_.

## Results

Of the 6112 patients that formed the primary efficacy population, 4951 provided SGRQ data that met the criteria for inclusion in the health outcomes population in 28 of 42 countries that participated in TORCH. Patients were allocated to one of five regions that had been pre-specified (see Additional file 1 for details). Baseline demographics by treatment group are presented in table 1, along with corresponding data from the patients in the ITT population [9]. Baseline variables were very similar across treatment groups and between the health outcomes and ITT populations. Differences between the two study populations in terms of proportion of patients recruited from different regions were due to the absence of suitably validated questionnaires for some languages in Asia-Pacific and Eastern Europe.

**Table 1:** Demographic and baseline characteristics of health outcomes population and all randomized patients (efficacy population)

Variable	Placebo (n = 1231)	SAL (n = 1232)	FP (n = 1248)	SFC (n = 1240)	Total HO population (n = 4951)	Total population (n = 6112)
Age at enrollment (years)	65.0 (8.2)	65.2 (8.2)	65.0 (8.5)	65.0 (8.3)	65.1 (8.3)	65.0 (8.3)
Male gender (%)	921 (75)	926 (75)	923 (74)	912 (74)	3682 (74)	4631 (76)
BMI (kg/m^2^)	25.8 (5.2)	25.7 (5.1)	25.6 (5.2)	25.6 (5.3)	25.7 (5.2)	25.4 (5.18)
Geographical region (%)						
USA	342 (27.8)	344 (27.9)	348 (27.9)	345 (27.8)	1379 (27.9)	1388 (22.7)
Asia-Pacific	89 (7.2)	93 (7.5)	95 (7.6)	93 (7.5)	370 (7.5)	758 (12.4)
Eastern Europe	185 (15)	186 (15.1)	185 (14.8)	184 (14.8)	740 (14.9)	1154 (18.9)
Western Europe	410 (33.3)	405 (32.9)	413 (33.1)	409 (33)	1637 (33.1)	1908 (31.2)
Other	205 (16.7)	204 (16.6)	207 (16.6)	209 (16.9)	825 (16.7)	935 (15.3)
Current smoker (%)	538 (44)	536 (44)	543 (44)	539 (43)	2156 (44)	2630 (43)
Pack-years smoked	49.5 (27.5)	50.6 (28.6)	50.0 (28.8)	47.7 (26.6)	49.4 (27.9)	48.5 (27.4)
Post-bronchodilator FEV_1 _(l)*	1.24 (0.42)	1.21 (0.40)	1.22 (0.42)	1.24 (0.43)	1.23 (0.42)	1.22 (0.42)
Post-bronchodilator FEV_1 _(% predicted*)	44.5 (12.3)	43.7 (12.4)	44.3 (12.3)	44.6 (12.3)	44.3 (12.3)	44.0 (12.4)
Reversibility (% predicted FEV_1_)*	3.7 (3.8)	3.7 (4.0)	3.6 (3.7)	3.7 (3.6)	3.7 (3.8)	3.7 (3.7)
Pre-bronchodilator FEV_1_/FVC ratio*	48.4 (11.0)	48.6 (11.0)	48.1 (10.8)	48.1 (10.8)	48.3 (10.9)	48.6 (10.8)
Baseline SGRQ total score	49.0 (17.4)	49.9 (16.6)	49.5 (17.1)	48.9 (17.4)	49.3 (17.1)	-

Data are mean (standard deviation) unless otherwise indicated

*Data are from visit one (screening)

BMI = body mass index; FP = fluticasone propionate; FEV_1 _= forced expiratory volume in one second; FVC = forced vital capacity; HO = health outcomes; SAL = salmeterol; SFC = salmeterol+fluticasone propionate; SGRQ = St George's Respiratory Questionnaire

More patients withdrew in the placebo arm than those receiving active treatment. By the end of 3 years, 45% of placebo-treated patients in this analysis had withdrawn compared with salmeterol 39%, FP 40% and SFC 35%.

### SGRQ change from baseline

**Figure 1:**
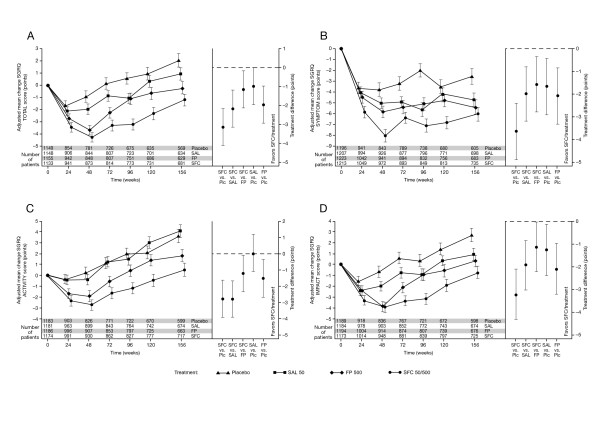
**Plots showing adjusted mean change for the SGRQ Total score (A) and the Symptoms (B), Activity (C) and Impacts (D) domains, over 3 years by treatment group**. A lower score indicates better health. The plot for each domain shows the change over time as the left-hand panel (error bars are standard error). The right-hand plots are from a repeated measures analysis of the effects of treatment over the 3 years of the study and are pair-wise comparisons between the treatment arms (error bars are 95% confidence intervals).

SGRQ scores by visit for the total and the domain scores are shown in Figure 1. The pattern of change in the placebo arm differed between domains: the improvement in Symptoms score over the first 6 months was sustained for the rest of the study; there was a small improvement in the Activity score over the first 6 months, thereafter it deteriorated; the Impacts domain showed an initial small improvement, followed by deterioration. Within domains, the pattern of change was similar in all treatment arms, but the magnitude differed. Averaged over the 3-year period, SFC was superior to placebo in all domains (all p < 0.001): Symptoms -3.6 (95% CI -4.8 to -2.4), Activity -2.8 (95% CI -3.9 to -1.6), Impacts -3.2 (95% CI -4.3 to -2.1). Salmeterol and FP showed an intermediate response. SFC was also superior to salmeterol (all domains p ≤ 0.001) and FP (all domains p < 0.05).

### Effect of region on SGRQ changes

**Figure 2:**
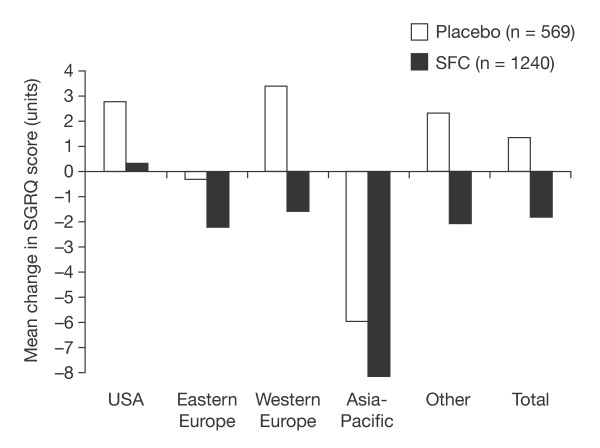
**Mean change in SGRQ Total score at 3 years by region for patients treated by placebo and SFC**. A lower score indicates better health. A test for an interaction between region and treatment effect was not significant (p = 0.16).

There were considerable regional variations in change in total SGRQ score (Figure 2). After 3 years, placebo-treated patients who completed the study were worse than at baseline in three regions, the USA, Western Europe and other, unchanged in Eastern Europe, and improved in Asia-Pacific. Numerically, patients receiving SFC improved in all regions except the USA. However, the actual treatment differences between SFC and placebo were fairly consistent, ranging from 1.8 to 5.0 at 3 years. A test for interaction between region and treatment effect was not significant (p = 0.16).

### SGRQ by GOLD stage

**Figure 3:**
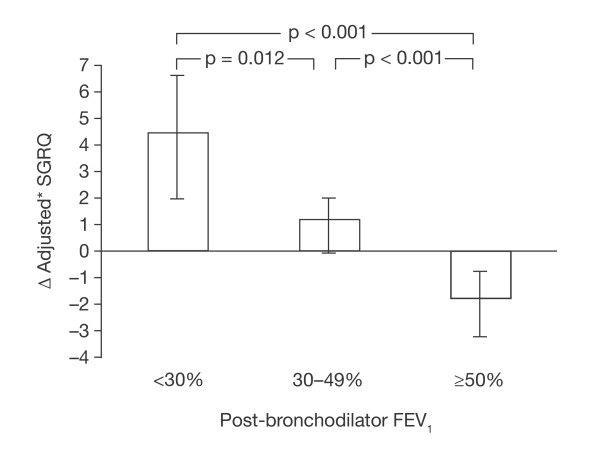
**Change in SGRQ Total score in patients categorized according to GOLD stage - patients treated with placebo only**. *Adjusted for baseline SGRQ, smoking, age, sex, BMI, region, and visit.

Baseline SGRQ scores in TORCH patients grouped by GOLD stage have been reported: GOLD stage II, 45.4 ± 17.7 (SD); GOLD stage III, 50.0 ± 16.5; GOLD stage IV, 56.5 ± 15.0 [12]. The differences between GOLD stage were clinically and statistically significant (p < 0.05), but within each stage patients exhibited a wide range in SGRQ score. Using MMRM analysis of change from baseline, the change over 3 years in patients treated with placebo was very different between GOLD stages (Figure 3). Patients in GOLD stage II who received placebo showed an overall improvement, while those in GOLD stages III and IV deteriorated. Changes with treatment have been reported elsewhere [12].

### Relationship between change in FEV_1 _and change in SGRQ score

For this analysis, data from all treatment arms were combined (3913 evaluable patients). In patients who withdrew early, the last value carried forward was used for both SGRQ and FEV_1 _data. The change in SGRQ at 3 years correlated significantly with change in FEV_1_: r = -0.24, p < 0.0001 (all treatment arms combined). In the regression between ΔSGRQ (dependent variable) and ΔFEV_1 _(independent variable), the intercept value for the SGRQ (i.e. the mean change in SGRQ that was associated with zero change in FEV_1_) was -0.7 units, p = 0.003. This indicates that the SGRQ score improved slightly overall, even in the absence of a change in FEV_1_.

Further analyses were performed to test for the influence of age, gender and region on the slope of this relationship and its intercept. When tested using FEV_1 _expressed in millilitres, women had a greater change in SGRQ score for a given change in FEV_1 _(p = 0.007), but this effect on the slope disappeared when change in FEV_1 _was expressed as percentage predicted (p = 0.7).

To test for effects of age, patients were divided into cohorts: < 55, 55 to 64, 65 to 74 and ≥ 75 years. In a univariate model, there was no significant effect on the slope (p = 0.027), but the intercepts did differ (p < 0.0001): < 55 years, -2.4 units; 55-64 years, -1.6 units; 65 to 74 years, 0.0 units; ≥ 75 years, +0.8 units, suggesting that in younger patients, SGRQ tended to improve, even in the absence of a change in FEV_1_.

**Figure 4:**
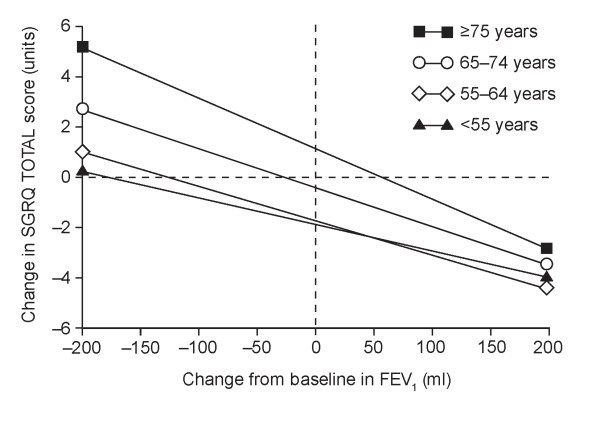
**Relationship between change in SGRQ Total score over the 3-year study period and change in FEV_1 _by age category**. A negative score indicates improved health. Using analysis of covariance: difference in slopes p = 0.008; difference in intercepts p < 0.0001.

In a multivariate model that adjusted for effects of sex, region, BMI, smoking status, baseline SGRQ, baseline FEV_1 _(as percentage predicted) and exacerbations in the previous year, age had an influence on both the slopes (p = 0.008) and intercepts (p < 0.0001) of the relationship between deterioration in SGRQ and decline in FEV_1 _(Figure 4). In this analysis, the intercepts were -1.9 units (<55 years), -1.7 units (55 to 64 years), -0.3 units (65 to 74 years) and +1.1 units (≥ 75 years). Older patients had a greater deterioration in SGRQ relative to the change in FEV_1 _than did patients < 64 years.

Region had no effect on the slope of the relationship between change in SGRQ and change in FEV_1 _(p > 0.05), but influenced the intercept (p < 0.0001): Asia-Pacific, -5.1 units; other, -1.2 units; Eastern Europe, -0.7 units; Western Europe, 0.0 units; USA, 0.1 units. No other covariate had a significant effect on this relationship in the univariate models.

### SGRQ and exacerbation rate

Data from all treatment arms were combined for this analysis. The change in SGRQ during the study was significantly related to exacerbation rate recorded during the study. In patients with no exacerbations, the SGRQ improved: -2.6 (95% CI -3.5 to -1.7) units/year; in patients with a low exacerbation rate (> 0 and ≤ 1 per year), there was a small overall improvement: -0.9 (95% CI -1.6 to -0.1) units/year; in patients with > 1 exacerbation per year, the SGRQ deteriorated: 2.8 (95% CI 2.1 to 3.6) units/year.

### Early withdrawal and SGRQ

**Figure 5:**
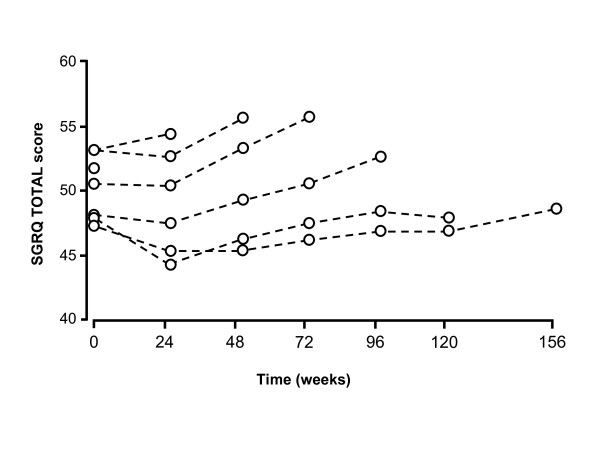
**Change in SGRQ Total score in patients treated with placebo**. Note: only 55% of patients remained in the study to 156 weeks.

There was a clear effect of both baseline SGRQ and rate of deterioration during the study on the likelihood of early withdrawal (Figure 5). Patients who entered the study with better health (average baseline score < 50) or did not deteriorate above this level, were more likely to remain in the study for more than 30 months. Drop-outs in the placebo arm up to week 156 varied across regions: Asia-Pacific 28%; Eastern Europe 35%, Western Europe 46%; other 50%; the USA 52%.

## Discussion

This study provides new insights into factors that determine health status decline and issues that must be considered in multinational studies that include health status as an outcome. There were improvements in all SGRQ domains with all active treatments, but SFC had the greatest effect. Interestingly, the pattern of change in score differed between domains. The Activity and Impact scores behaved similarly to those reported in ISOLDE, another 3-year study [8] but the behaviour of the Symptoms domain was clearly different because the initial improvement was maintained for the rest of the study with no apparent deterioration. A similar pattern has been reported with the SGRQ symptoms score in the 3-year Bronchitis Randomized on NAC Cost-Utility Study (BRONCUS) trial of n-acetyl cysteine [13]. One possible explanation may be the large and prolonged effect of a single exacerbation on SGRQ score, particularly the Symptoms domain, which may continue for over 3 months [14]. In TORCH, patients were excluded only if they had an exacerbation requiring treatment during the 2-week run-in period, so the sustained improvement seen in this domain may have occurred if some patients had an exacerbation in the weeks before run-in. In BRONCUS, exacerbations prior to randomization were not an exclusion criterion. By contrast, in ISOLDE, the patients had a 6-week run-in period and were then given a 2-week course of prednisolone, so effects of any prior exacerbations would have been minimized.

Our exploratory subgroup analyses provide observations that generate new hypotheses about health status decline in COPD. The rate of deterioration in men and women relative to loss of FEV_1 _appeared to be the same once gender differences in the absolute value of FEV_1 _were taken into account. Patients with severe and very severe airway obstruction at baseline showed significant deterioration in their health status over 3 years, whereas those with moderate obstruction improved. One possible explanation for this difference is the effect of exacerbations on SGRQ scores [15,16], and the known relationship between FEV_1 _and exacerbation rates [13,14,17]. In TORCH, patients with no exacerbations showed a mean improvement in Total SGRQ score over 3 years, whereas those with > 1 exacerbation per year had a significant worsening of health. A link between SGRQ deterioration and exacerbation rate was reported in ISOLDE, and statistical modelling of those data suggested a causal relationship between a lower rate of exacerbations in patients with FP and a slower rate of worsening of SGRQ [16]. These observations from ISOLDE and TORCH suggest that recurrent exacerbations have a cumulative effect on health status similar to that reported for FEV_1 _[18,19].

Older people appeared to have a faster loss of health status than younger people. This was seen after other demographic and disease-related effects such as gender and baseline FEV_1 _had been taken into account, suggesting that health status deterioration in COPD may accelerate with age. This may be related to increasing comorbidity, but another factor may be an age-related increase in frailty and self-reported functional decline, rather than a specific chronic disease effect [20].

There appears to have been a 'trial effect' in Asia-Pacific, where sustained improvements in SGRQ score were seen irrespective of whether patients received active study treatment. One plausible explanation for this is that patients' health in that region, particularly China, may have improved because they received better healthcare by joining a clinical trial (JP Zheng, personal communication). China contributed 65% of the Asia-Pacific patients and a similar SGRQ improvement has been reported in the placebo limb of two large studies from that country [21,22], although this was not seen in a third [23]. One of the studies compared SFC with placebo and the active treatment produced a significantly greater effect on SGRQ despite a large effect on placebo [22]. In TORCH, the relationship between deterioration in SGRQ and decline in FEV_1 _in Asia-Pacific was not different from that seen in the other regions. Taken together, these observations suggest that the SGRQ does measure treatment effects and disease progression in China and Asia-Pacific in a similar way to other countries. However, such instruments also appear to detect health status gains that may occur on recruitment to a clinical trial in developing health economies. In this context, it should be noted that the withdrawal rate in the USA was 56% compared with 29% in Asia-Pacific.

TORCH extends the observations reported in other studies that patients with poor health at baseline and those that deteriorated faster were more likely to withdraw earlier than others [8,24]. In TORCH, the only patients who remained in the study for more than 30 months were those in whom there had, on average, been no deterioration in health status compared with baseline. This suggests that patients and their physicians expect the patient's health to improve on entering a study of this kind, and any deterioration may lead to early withdrawal. This will lead to a 'healthy survivor' effect as many of the sickest people withdraw. That effect becomes especially important when there is differential drop-out between treatment arms, as in this study where there was a 10% absolute and 20% relative difference in drop-out rate between the placebo and SFC arms. This 'informative drop-out' process may lead to a biased estimate of treatment efficacy, in this case an underestimate. Health status measurements now form an established assessment of treatment efficacy in COPD because they are a marker of an important clinical outcome (health-related quality of life) and are poorly correlated with FEV_1_. The observations made here were obtained with the SGRQ but are likely to be seen with any validated health status measure in COPD, as a comparison between the SGRQ and a new instrument with a very different structure showed that the two questionnaires appear to be highly correlated and behave in a very similar way [25]. This analysis suggests that studies with a low baseline exacerbation frequency, different drop-out rates, and large Eastern Europe and Asia-Pacific region participation may not give the same results as those involving participants in Western Europe and the USA.

## Competing interests

All authors have completed the Unified Competing Interest form at http://www.icmje.org/coi_disclosure.pdf (available on request from the corresponding author) (URL) and declare; **P.W.J**. has received consulting fees from Almirall, AstraZeneca, GlaxoSmithKline, Novartis, Roche and Spiration; speaking fees from AstraZeneca and GlaxoSmithKline; and grant support from GlaxoSmithKline. **J.A.A**. is employed by and holds stock in GlaxoSmithKline. **P.M.A.C**. has received consulting fees from AstraZeneca, GlaxoSmithKline, Novartis, Nycomed and Pfizer; speaking fees from GlaxoSmithKline and Nycomed; and grant support from Boehringer-Ingelheim and GlaxoSmithKline. **B.R.C**. has received consulting fees from Altana, AstraZeneca, Boehringer-Ingelheim and GlaxoSmithKline; speaking fees from Altana, AstraZeneca, Boehringer-Ingelheim and GlaxoSmithKline; and grant support from Boehringer-Ingelheim and GlaxoSmithKline. **G.T.F**. has received consulting fees from Astra Zeneca, Boehringer-Ingelheim, GlaxoSmithKline, Novartis and Pearl Therapeutics; speaking fees from Boehringer-Ingelheim, GlaxoSmithKline and Pfizer; and grant support from Boehringer-Ingelheim, Forest, GlaxoSmithKline and Novartis. **C.J**. has received consulting fees from Altana, AstraZeneca, Boehringer-Ingelheim, GlaxoSmithKline and Novartis; speaking fees from Altana, AstraZeneca, Boehringer-Ingelheim, GlaxoSmithKline and Novartis; and grant support from GlaxoSmithKline. **J.C.Y**. is employed by and holds stock in GlaxoSmithKline. **J.V**. has received consulting fees from AstraZeneca, Boehringer-Ingelheim, GlaxoSmithKline, Hoffman-LaRoche and Nycomed; speaking fees from AstraZeneca, Boehringer-Ingelheim and GlaxoSmithKline; and grant support from GlaxoSmithKline. **M.D.S**. was employed by GlaxoSmithKline when the study was conducted and during manuscript preparation, and holds stock in GlaxoSmithKline, Elan Pharma Ltd and Janssen Cilag Ltd.

## Authors' contributions

PWJ, PMAC, JAA, BRC, GTF, CJ, JCY and JV contributed to the initiation, design, and conduct of the study, the interpretation of data, and manuscript development; MDS contributed to the interpretation of data and manuscript development; JAA designed and performed the statistical analyses. All authors have seen and approved the final submitted version of the manuscript.

## Supplementary Material

Additional file 1 **The number of patients with at least one valid SGRQ in which a total score could be calculated completed in each country**. Lists the number of patients with at least one valid SGRQ in which a total score could be calculated completed in each country. Click here for file
