# Supplementary material for: Health status in the TORCH study of COPD: treatment efficacy and other determinants of change
Source: Respir Res. 2011 May 31;12(1):71. doi: 10.1186/1465-9921-12-71 (PMC3117702; doi:10.1186/1465-9921-12-71)
Supplement: Additional file 1 — The number of patients with at least one valid SGRQ in which a total score could be calculated completed in each country. Lists the number of patients with at least one valid SGRQ in which a total score could be calculated completed in each country. [file 1465-9921-12-71-S1.DOCX]

**ONLINE DATA SUPPLEMENT**

The number of patients with at least one valid SGRQ in which a total score could be calculated completed in each country.

**Asia-Pacific**: China (n = 240), Hong Kong (n = 85), Singapore (n = 45).

**Eastern Europe**: Czech Republic (n = 181), Estonia (n = 151), Hungary (n = 142), Poland (n = 130), Russia (n = 58), Slovakia (n = 78).

**USA** (n = 1379).

**Western Europe**: Belgium (n = 68), Denmark (n = 125), Finland (n = 176),
France (n = 232), Germany (n = 147), Greece (n = 176), Italy (n = 54), the Netherlands (n = 109), Norway (n = 163), Spain (n = 246), the UK (n = 141).

**Other**: Argentina (n = 60), Australia (n = 179), Brazil (n = 51), Canada (n = 159), Mexico (n = 72), New Zealand (n = 128), South Africa (n = 176).
